# Bexarotene combined with lapatinib for the treatment of Cushing’s disease: evidence based on drug repositioning and experimental confirmation

**DOI:** 10.1038/s41392-020-00284-7

**Published:** 2020-08-29

**Authors:** Haoying Yu, Shuyue Ren, Jingrong Wang, Tingting Lv, Lan Sun, Guanhua Du

**Affiliations:** 1 Institute of Materia Medica, Chinese Academy of Medical Science and Peking Union Medical College, 1 Xian Nong Tan Street, 100050 Beijing, China; 2 Beijing Key Laboratory of Drug Targets Identification and Drug Screening, 100050 Beijing, China; 3 The State Key Laboratory of Bioactive Substance and Function of Natural Medicines, 1 Xian Nong Tan Street, 100050 Beijing, China

**Keywords:** Endocrine cancer, Endocrine cancer, Target validation

**Dear Editor,**

Cushing’s disease (CD) is a rare disease manifested as Cushing’s syndrome caused by adrenocorticotropic hormone (ACTH) consistently over-secreted by adrenocorticotropic adenomas, followed by stimulation of the adrenal gland to secrete considerable cortisol, triggering metabolic dysfunction, and leading to death from complications. However, pituitary adrenocorticotropic adenoma-directed drugs can only inhibit ACTH secretion or hamper tumour growth, which limits their clinical applications. Recently, Nur77 (also known as NR4A1 and NGFI-Ba, a kind of nuclear receptor), an important positive transcription regulator of pro-opiomelanocortin (POMC), the precursor of ACTH, has been regarded as a promising CD target.^1^ Hence, it would be promising to identify a combination treatment that targets Nur77 and another CD target to cover hormone normalisation in the short term and tumour suppression or even elimination in the long term.

De novo drug synthesis or the random combination of two drugs is a time-consuming and costly process. More recently, drug repositioning or repurposing, which refers to the use of marketed drugs or drug combinations for new indications, has recently gained popularity as an alternative strategy.^2^

In our present study, we repositioned the Food and Drug Administration (FDA)-approved bexarotene (BEXA), which is approved for the therapy of cutaneous T-cell lymphoma, to determine a new indication for CD.^3^ First, we constructed a protein-protein interaction network centred on Nur77 with the STRING platform, and RXRα was selected (Fig. 1a, Supplementary Fig. S1a and Supplementary Table S1). When the CD target “RXRα” was input into the DrugBank database, approved targeting RXRα drugs were found, of which BEXA had the highest score in the analysis using WebGestalt platform (Supplementary Table S2 and Supplementary Fig S1b), followed by the in silico proteome-wide prediction of targets interacting with BEXA using the Ligand Express platform provided by Cyclica (Supplementary Table S4) and molecular docking analysis between BEXA and RXRs using a CHARMM-based powerful docking method named CDOCKER (Supplementary Fig. S2 and Supplementary Table S3).

**Fig. 1:**
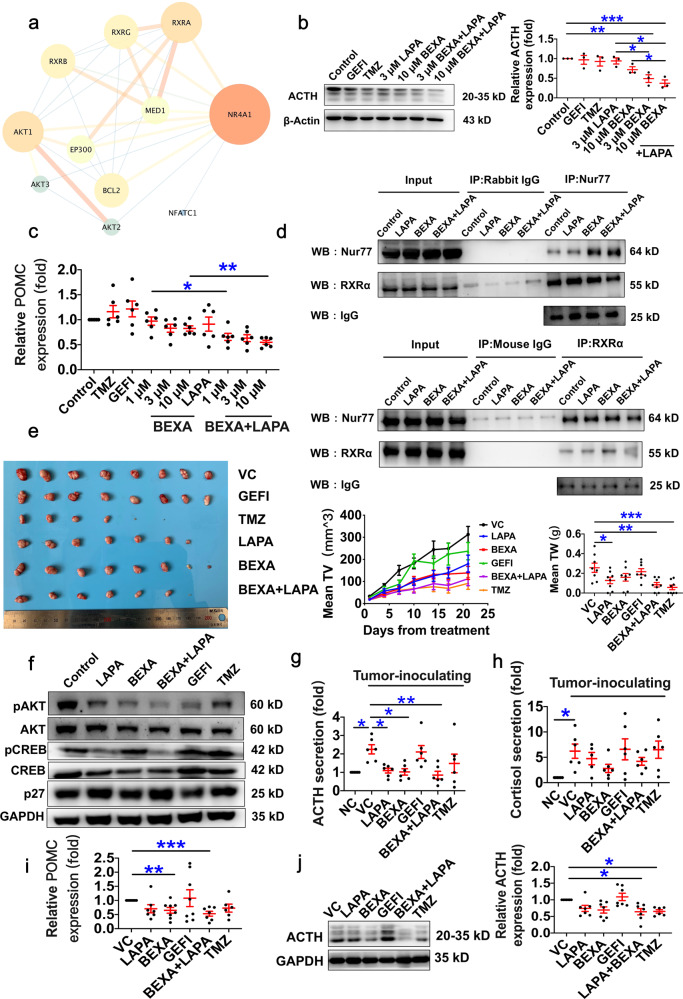
Synergistic inhibition of BEXA/LAPA on pituitary adenoma growth and ACTH synthesis via computational drug repositioning and experimental confirmation. **a** Direct interaction module obtained from the Nur77-centred PPI network. Line thicknesses and colours represent evidence of the PPI, and node size and colours represent the degree. **b**, **c** The combination treatment of BEXA and LAPA reduced ACTH and POMC expression in AtT-20 cells. Cells were seeded onto 100 mm dishes at a concentration of 5 × 10^5^ cells/dish. After adhering to the dish surface (24 h later), AtT-20 cells were treated with the indicated drugs (BEXA, bexarotene; LAPA, lapatinib; GEFI, gefitinib; and TMZ, temozolomide). **b** Forty-eight hours later, the expression levels of ACTH in AtT-20 cells were detected using Western blot analysis. **c** Six hours after the cells were treated with the indicated drugs, the relative expression levels of POMC mRNA to β-actin mRNA were detected using RT-qPCR. Control indicates cells treated with the vehicle. *p < 0.05, **p < 0.01, ***p < 0.001 vs. control, LAPA or BEXA. The experiment was repeated three times. Every time, three dishes were used in each group. **d** A co-immunoprecipitation assay was applied to detect Nur77-RXRα complex formation. Quiescent AtT-20 cells were incubated with 10 μM BEXA, 10 μM LAPA or 10 μM BEXA + 10 μM LAPA for 24 h. As shown in the upper panel, cell lysates were immunoprecipitated with anti-Nur77 resin and then immunoblotted with RXRα and Nur77 antibodies. As shown in the lower panel, cell lysates were immunoprecipitated with anti-RXRα resin and then immunoblotted with RXRα and Nur77 antibodies. Mouse IgG and rabbit IgG was used as a negative control. **e**–**j** Synergistic effects of BEXA and LAPA on inhibiting tumour growth in vivo. Forty-eight tumour-bearing mice that met the standard (with tumour diameters reaching 3–5 mm) were divided into six groups: vehicle control group (treated with 0.5% CMC-Na), GEFI group (treated with gefitinib, 75 mg/kg), TMZ group (treated with temozolomide, as a positive control, 20 mg/kg), LAPA group (treated with LAPA, 30 mg/kg), BEXA group (treated with BEXA, 50 mg/kg) and the combination group (LAPA, 30 mg/kg; BEXA, 50 mg/kg). Normal control group indicates sham mice treated with 0.5% CMC-Na. *n* = 8 per group. The left panel shows representative tumour tissues of animals at the end of treatment, and no tumour tissue indicates that the tumour bulk was completely surpassed. **h** Diameters of the major and minor axes were measured every three days (middle panel). Tumour volume (TV) = 1/2 × diameter of the major axis × diameter of the minor axis^2^. Tumours were weighed at the end of treatment (right panel), TW tumour weight. **f** The expression levels of p27 and the phosphorylation levels of Akt and CREB in tumour tissues were detected using Western blot. The secretion levels of ACTH (**g**) and cortisol (**h**) in the plasma were detected using an enzyme-linked immunosorbent assay. *n* = 6. **i** The relative mRNA expression levels of POMC in the tumours were detected using RT-qPCR. β-Actin mRNA was used as a loading control. **j** The relative expression levels of ACTH in the tumours were detected using Western blot (*n* = 6–8). NC normal control, sham mice treated with the vehicle. VC vehicle control, refers to tumour-bearing mice treated with the vehicle. *p < 0.05, **p < 0.01, ***p < 0.001 vs. VC

Second, another FDA-approved targeted EGFR drug, lapatinib (LAPA), can be combined with BEXA to treat CD. Based on Kyoto Encyclopaedia of Genes and Genomes pathway analysis, the PI3K-AKT pathway was found to be downstream of both RXRα and Nur 77, indicating that the combination of BEXA and another drug that targets the PI3K-Akt signalling pathway may exert a crosslinked effect (Supplementary Fig. S3 and Supplementary Table S5). As one of the upstream regulators of AKT, “EGFR” was input as a target in the DRUGBANK database, and then the approved EGFR-targeting drugs were selected (Table S6), of which LAPA was confirmed according to its IC_50_ on AtT-20 cell survival (Supplementary Table S7).

Third, the synergistic inhibition of BEXA/LAPA on ACTH generation and on adrenocorticotropic adenoma growth by promoting the formation of the Nur77-RXRα dimer was confirmed both in vitro and in vivo. BEXA combined with LAPA has a synergistic inhibitory effect on pituitary adenoma cell growth. The combination index (CI) of BEXA/LAPA was calculated and indicated synergistic inhibition on AtT-20 cell proliferation (CI < 1, Supplementary Fig. S4a and Supplementary Table S8) via inactivation of the PI3K-Akt signalling pathway, which was confirmed by a reduction in the EdU incorporation rate (Supplementary Fig. S4b), decreased phosphorylation levels of Akt, CREB, JNK, and cJun and upregulated expression levels of p27 (Supplementary Fig. S4c, d). Then, the synergistic inhibitory effect of BEXA and LAPA on the expression of ACTH (Fig. 1b) and the POMC (Fig. 1c), which is the precursor of ACTH in AtT20 cells were observed. Accordingly, we found that BEXA and LAPA enhanced the formation of the Nur77-RXRα dimer, a critical process that regulates POMC transcription (Fig. 1d).

The combination of BEXA/LAPA synergistically inhibited pituitary adenoma growth and ACTH production in vivo. The mouse adenoma cell line AtT-20 was inoculated subcutaneously into BALB/c mice to develop implanted tumours. The treatment of tumour-bearing mice with the combination of LAPA/BEXA, as well as each drug alone, decelerated tumour growth, while the inhibitory effect of the combination of BEXA/LAPA was comparable to that of the positive control (Temozolomide, Fig. 1e). The phosphorylation of proteins in the PI3K-Akt-CREB cell signalling transduction pathway also changed in a manner similar to that detected in vitro (Fig. 1f). The hormone expression levels in the plasma and tissues were also measured to confirm the hormone-regulating functions of the drugs in vivo, which were evidenced by a reduction in the expression levels of ACTH and its downstream hormone cortisol in the plasma (Fig. 1g, h) which is confirmed by the changes in the expression levels of ACTH and POMC in tumours (Fig. 1i, j). There was no change in the expression levels of corticotrophin-releasing hormone (CRH), an upstream hormone of ACTH (data not shown), indicating that the effect of BEXA and LAPA on ACTH is hypothalamus independent.

In fact, the role of RARα agonists in CD therapy has been debated: early findings suggest that agonists of RXR, such as retinoic acid, suppress ACTH secretion in Cushing’s disease via inhibition of the transcriptional activity of AP-1, Nur77, and Nurr1,^4^ while this seems to be incorrect according to later experiments on another synthetic RARα agonist, Am80, which was found to increase POMC mRNA expression, CRH-induced ACTH secretion, and POMC promoter activity.^5^ In the present study, we confirmed the role of the RXRα agonist BEXA on ACTH secretion and showed the synergistic inhibition of BEXA/LAPA on pituitary adenoma growth and ACTH synthesis, which are associated with hormone normalisation and tumour suppression, via computational drug repositioning and experimental confirmation. The major findings are that, first, we repositioned BEXA for the treatment of CD via artificial intelligence prediction and selected the BEXA/LAPA combination based on computational cell signalling transduction analysis. RXRα and its ligand BEXA, which were approved by the FDA and European Medicines Agency for the treatment of cutaneous T-cell lymphoma in 1999 and 2001, were selected as candidates that are also financially attractive. Second, we observed the synergistic inhibition of BEXA/LAPA on ACTH production and on pituitary adenoma growth via crosstalk between RXRα and the EGFR downstream PI3K/AKT cell signalling pathway, which is shown in Fig. S6 as follows: LAPA inhibits the phosphorylation of EGFR and HER2 and blocks a series of downstream proteins, eventually leading to the inhibition of tumour formation and AtT-20 cell proliferation. On the one hand, LAPA inactivates the EGFR/PI3K/Akt signalling pathway and then inhibits JNK-cJun, hampering RXRα phosphorylation or mutation and hence promoting the pharmacological effects of BEXA on RXRα. On the other hand, BEXA actives RXRα to form the heterodimer RXRα-Nur77, thereby reducing the quantity of Nur77 available to bind to the POMC promoter and consequently suppressing POMC expression and the reduction in ACTH.

In conclusion, our present study revealed a novel combination therapy of BEXA/LAPA through a Nurr77-dependent mechanism via drug repositioning and experimental confirmation, which provides new ideas for CD treatment, especially for the treatment of EGFR/HER2-positive patients since LAPA is an EGFR/HER2 inhibitor.

## Supplementary information

Supplemental materials
