# Supplementary material for: Bexarotene combined with lapatinib for the treatment of Cushing’s disease: evidence based on drug repositioning and experimental confirmation
Source: Signal Transduct Target Ther. 2020 Aug 29;5:175. doi: 10.1038/s41392-020-00284-7 (PMC7456421; doi:10.1038/s41392-020-00284-7)
Supplement: Supplementary file 1 — Supplemental materials [file 41392_2020_284_MOESM1_ESM.docx]

Supplementary Materials

**Title:** Bexarotene Combined with Lapatinib for the Treatment of Cushing’s Disease: Evidence Based on Drug Repositioning and Experimental Confirmation

**Authors:** Haoying Yu^1,2^, Shuyu Ren^1,3^, Jingrong Wang ^1,3^, Tingting Lv^1,2^, Lan Sun ^1,2,3^*and Guanhua Du ^1,2,3*^

1. Institute of Materia Medica, Chinese Academy of Medical Science and Peking Union Medical College,1 Xian Nong Tan Street, Beijing 100050, China.

2. The State Key Laboratory of Bioactive Substance and Function of Natural Medicines 1 Xian Nong Tan Street, Beijing 100050, China

3. Beijing Key Laboratory of Drug Targets Identification and Drug Screening Beijing 100050, China.

*: Corresponding Authors: Prof. Lan Sun; E-mail: [sunhanxing2005@imm.ac.cn; Prof](mailto:sunhanxing2005@imm.ac.cn;%20Prof). Guanhua Du; E-mail:dugh@imm.ac.cn

Additional email for co-authors: [yhy@imm.ac.cn](mailto:yhy@imm.ac.cn) to Haoying Yu; [renshuyue@imm.ac.cn](mailto:renshuyue@imm.ac.cn) to Shuyue Ren; [wangjingrong@imm.ac.cn](mailto:wangjingrong@imm.ac.cn) to Jingrong Wang. lvtingting@imm.ac.cn to Tingting Lv.

**This file includes:**

**Materials and methods, Supplemental Figures and Supplemental Tables**

**Supplemental Figures:**

**Figure S1. RXRα and AKT were selected as targets through PPI analysis centered by Nur77.**

**Figure S2. The interactions** **between BEXA and RARs.**

**Figure S3. PPI network analysis centered on RXRα.**

**Figure S4. BEXA and LAPA synergistically inhibit AtT20 cell proliferation via the inactivation of the PI3K-AKT signaling pathway.**

**Figure S5. The ACTH expression and secretion levels and the POMC expression levels in AtT20 cells in response to BEXA in a concentration dependent manner.**

**Figure S6. Mechanism of synergistic inhibitory effects of LAPA and BEXA on ACTH production and on pituitary adenoma cell proliferation.**

**Supplemental Tables:**

**Table S1. Top 10 scored hub proteins in the Nur77 (NR4A1) PPI network.**

**Table S2. Approved drugs targeting RXRα.**

**Table S3. The -docking energy between BEXA and RAR family proteins**

**Table S4. Top 22 scored proteins predicted to interact with BEXA in the CD protein network.**

**Table S5. KEGG enrichment analysis of genes of interest.**

**Table S6. Drugs targeting EGFR.**

**Table S7. IC50s of bexarotene and lapatinib.**

**Table S8. Combination index of bexarotene and lapatinib.**

**Materials and methods**

Databases and platforms

PPI network analysis was conducted by Search Tool for the Retrieval of Interacting Genes (STRING; http://string-db.org) (version 11.0) and diagrams were produced using Cytoscape. Drug enrichment analysis was executed on WEB-based GEne SeT AnaLysis Toolkit (WebGestalt; http://www.webgestalt.org/option.php) (version 2019) platform based on DrugBank and GLAD4U databases. KEGG pathway analysis was conducted through The Database for Annotation, Visualization and Integrated Discovery (DAVID;http://david.ncifcrf.gov) (version 6.8) based on Kyoto Encyclopedia of Genes and Genomes (KEGG) database. Information of drugs directed to specific targets were collected through DrugBank database. (https://www.drugbank.ca/). Prediction of targets interacted with BEXA was implemented on Ligand Express platform provided by CYCLICA Inc.

Molecular docking

Molecular docking performed using Discovery Studio 2016 software (BIOVIA, CA, US ) based on protein data bank (PDB）and (Pubchem).

The crystal structure of RAR α, RAR β, RAR γ, PPAR α, PPAR δ and PPAR γ complexed with the inverse agonist BMS493 and a corepressor fragment, selective partial agonist BMS641 [3-chloro-4-[(E)-2-(5,5-dimethyl-8-phenyl-5,6-dihydronaphthalen-2-yl)ethenyl]benzoic acid], trifarotene (CD5789), agonist AZ 242, a phenoxyacetic acid partial agonist and T2384, respectively, were retrieved from the Protein Data Bank (PDB ID: 3kmz, 4jyi, 6FX0, 1i7g, 3PEQ and 3k8s). Wherein the resolutions of protein structures were 2.1 Å, 1.9 Å, 1.9 Å, 2.2 Å, 2.4Å and 2.55 Å, separately.

Preparation of compound and proteins Bexarotene was prepared with prepare ligands module of small molecules. For the preparation of proteins, the water and the co-crystallized complex were removed, and hydrogens atoms were added, generating valid single 3D conformation by means of washing and energy minimizing. The RAR α, RAR β, RAR γ, PPARα, PPAR δ and PPAR γ proteins were then further processed with the prepare protein module to model missing loop regions, calculate protein ionization and protonate the protein structure. The prepared proteins were defined as the receptors and the binding site were defined from PDB site records with the define and edit binding site module. Finally, the binding site sphere of 3kmz, 4jyi, 6FX0, 1i7g, 3PEQ and 3k8s were selected separately for molecular docking analysis.

In the CDOCKER modules, we set the hearting steps, hearting target temperature, cooling steps, cooling target temperature and Forcefield to 2000，700，5000，300和CHARMm, separately. Bexarotene was docked into the binding site by utilizing the CDOCKER modules.

Reagents

Murine pituitary corticotrophin tumor AtT20 cells (ATCC CRL-1795) and HEK293T cells were obtained from the ATCC (ATCC, VA, USA). Dulbecco's modified Eagle medium (DMEM) and fetal bovine serum (FBS) were purchased from Thermo Fisher Scientific (Thermo, MA, USA). The penicillin/streptomycin was from Solarbio (Solarbio, Beijing, CHN). Lapatinib, gefitinib, and temozolomide were purchased from Selleck Chemical (Selleck, TX, USA). Bexarotene was provided by Dr. Lv Yang, Beijing Key Laboratory of Polymorphic Drugs, Center of Pharmaceutical Polymorphs.

Cell culture and proliferation assay

AtT20 and HEK293T cells were cultured in DMEM supplemented with 10% FBS, 100 IU/mL penicillin and 100 μg/mL streptomycin. Cells were cultured in a humidified incubator at 37°C with 5% CO_2_.

AtT20 cells were seeded in 96-well plates (2000 cells per well) and incubated with drugs at appropriate concentrations for 72 hours. Then the cell numbers were counted using a Cell Counting Kit-8 (KeyGen Biotech, Nanjing, Jiangsu, CHN) according to the instruction of manufacturer at the absorbance of 450 nm. Combination index (CI) of two drugs was calculated as method introduction by Chou and Talalay.

Cell proliferating activity was detected using EdU Cell Proliferation Kit for Imaging (KeyGen Biotech, Nanjing, Jiangsu, CHN) according to the instruction.

Animals and tumor formation

All experiments were approved by the Animal Studies Committee of Peking Union Medical College.Seven to eight-week-old female BALB/c mouse were bred at Charles River (Charles River Laboratories, Beijing, CHN). Adopt the mice to the environment for 24 *hs* and removed hair under the armpits with small animal shavers and hair removal cream. Mouse was inoculated with AtT-20 cells (5×10^5^ cells per mice, 0.2 mL of suspension) in logarithmic growth phase. About 5 days after inoculation, tumor diameters reached 3-5 mm deemed as qualified models and they were grouped randomly and administrated with different treatment for next 21 days. Body weight and tumor volumes of animals were measured every 3 days, tumor volumes were measured with a caliper and calculated as π/6×large diameter× small diameter^2^, as previously described.^24^ On the last treatment day (day 21), blood was collected with EDTA tubes and tumors, spleens as well as thymus were excised and weighed.

Measurement of hormones concentration

Protein of cells, blood samples were collected after different treatments for the measurement of ACTH concentration. Adrenal, hypothalamus and pituitary were extracted and frozen in liquid nitrogen immediately and were ground with tissue homogenizer to extract the proteins followed by the detection of cortisol and CRH. The ACTH, cortisol and CRH expression levels in cells, blood and tissues were detected using ELISA assay according to the manufacturer's instructions immediately (CUSABIO, Wuhan, Hubei, CHN).

Western blot analysis

The AtT-20 cells and tumors were lysed using the radio immunoprecipitation assay (RIPA) lysis buffer (Solarbio, Beijing, CHN) containing protease inhibitor and phosphatase inhibitor cocktails. The anti-phospho-CREB, anti-CREB, anti-phospho-cJun, anti-cJun, anti-phospho-JNK, anti-phospho-Akt (Cell Signaling, Danvers, MA, USA), anti-Nur77, anti-p27/Kip1, (Abcam, Cambridge, MA, USA), anti-GAPDH (Proteintech, Wuhan, Hubei, China), anti-Akt, anti-JNK, anti-ACTH/POMC (Santa cruz, CA, USA) and anti-RXRα (Thermo, MA, USA) antibodies were used as the primary antibodies. The signals were detected using the enhanced chemiluminescence detection method and were quantified by densitometry.

RNA isolation and quantitative real-time PCR

The total RNA of AtT20 cells were isolated using Trizol (Thermo, MA, USA) according to the manufacturer's protocol. The quality and quantity of RNA was measured by a Nanodrop Thermo, MA, USA). A260/A280 of RNA samples approximately 2.0 were qualified for the gene expression analysis. 0.5 μg of RNA was used as a template for synthesizing cDNA with Prime Script RT reagent Kit (TaKaRa, Beijing, CHN) according to the manufacturer's instruction. TB Green Premix Ex Taq ii (TaKaRa, Beijing, CHN) were used to perform quantitative real-time PCR to assay the expression levels of POMC (NM_008895.3) mRNA transcripts. The following primer sequences were used: mouse POMC (forward, 5'-CAGTGCCAGGACCTCACC-3', reverse, 5'-CAGCGAGAGGTCGAGTTTG-3') and mouse β-actin (forward, 5'-AGGCCAACCGTGAAAAGATG-3'; reverse, 5'-TGGCGTGAGGGAGAGCATAG-3'). β-actin was used as a reference gene to standardize gene-expression levels. Results are expressed as ratio of POMC and β-actin.

Co-immunoprecipitation assay

For co-immunoprecipitation assay, Co-Immunoprecipitation Kit (Thermo, MA, USA) was used according to the instruction. Briefly, Anti-mouse primary antibody to RXRα (Thermo, MA, USA) and anti-rabbit primary antibody to Nur77(Abcam, Cambridge, MA, USA) were fixed by incubation with protein A/G agarose for 2 hours at room temperature. Normal mouse IgG and rabbit IgG were incubated as non-specific control. Proteins (antigens)- antibody- agarose complex was formed by incubating antibody-agarose with the supernatants overnight at 4°C. Proteins-antibody was made to dissociate with agarose and heated for 5 minutes at 100°C with loading buffer. Precipitated protein complexes were separated by SDS-PAGE as described above. Blots for RXRα were incubated with anti- rabbit primary RXRα antibody (Proteintech, Wuhan, Hubei, China) to avoid interference of heavy chain of fragmented antibody at 55kD.

**Supplemental Figures and Figure legends**

**Figure S1**

**
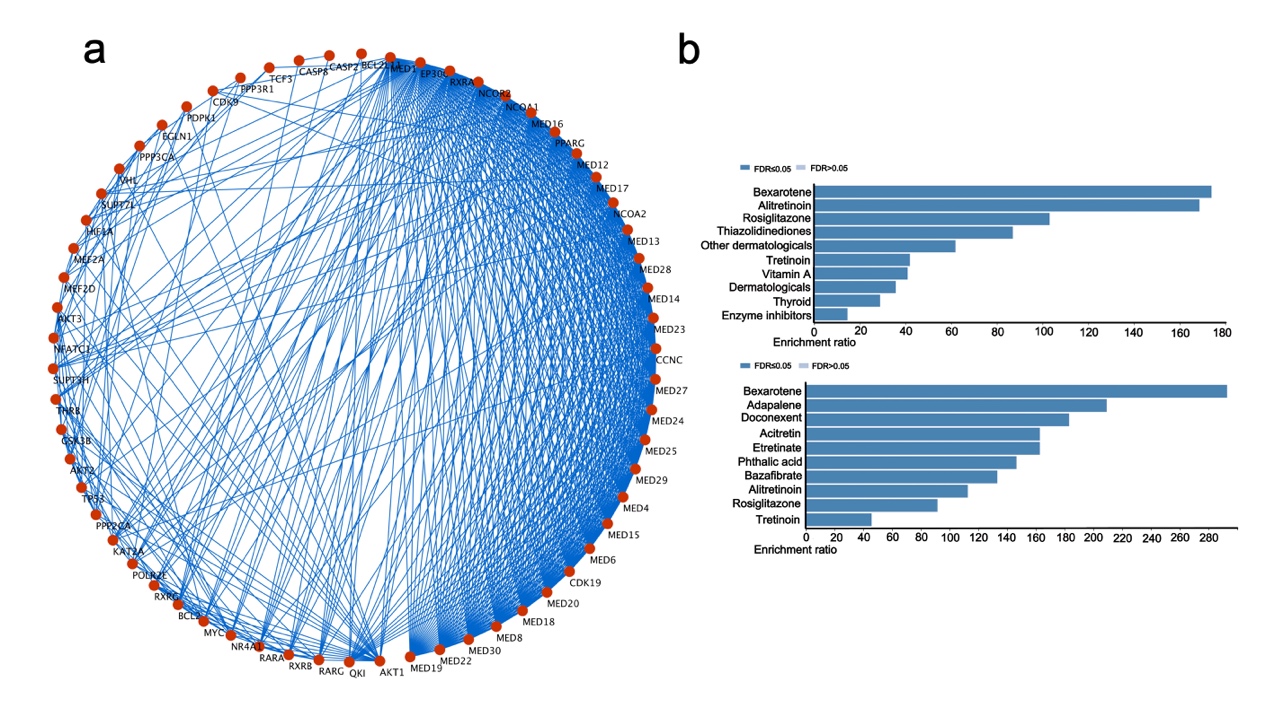
**

**Fig S1. RXRα and AKT were selected as targets through the PPI network analysis centred on Nur77.** a: Result of the Nur77 related PPI network analysis. The minimum required interaction score was 0.9, and the maximum number of interactors to show was 50. b: Enrichment results of the hub proteins revealed by setting GLAD4U (the upper panel) and DRUGBANK (the down panel), which were utilized as functional database using WebGestalt platform.

**Fig S2**


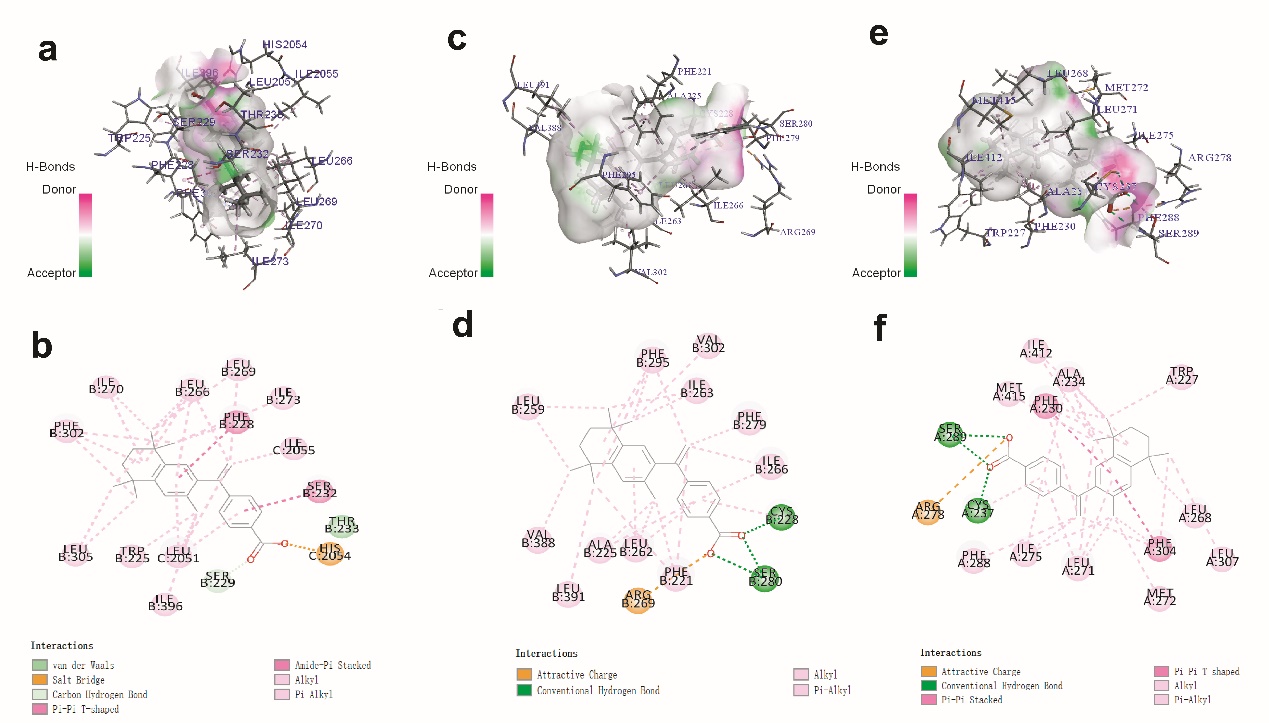


**Fig S2. The interactions between BEXA and RARs.** a-b: The docking mode between BEXA and RARα; c-d: The docking mode between BEXA and RARβ; e-f: The docking mode between BEXA and RARγ.

**Fig S3**

**
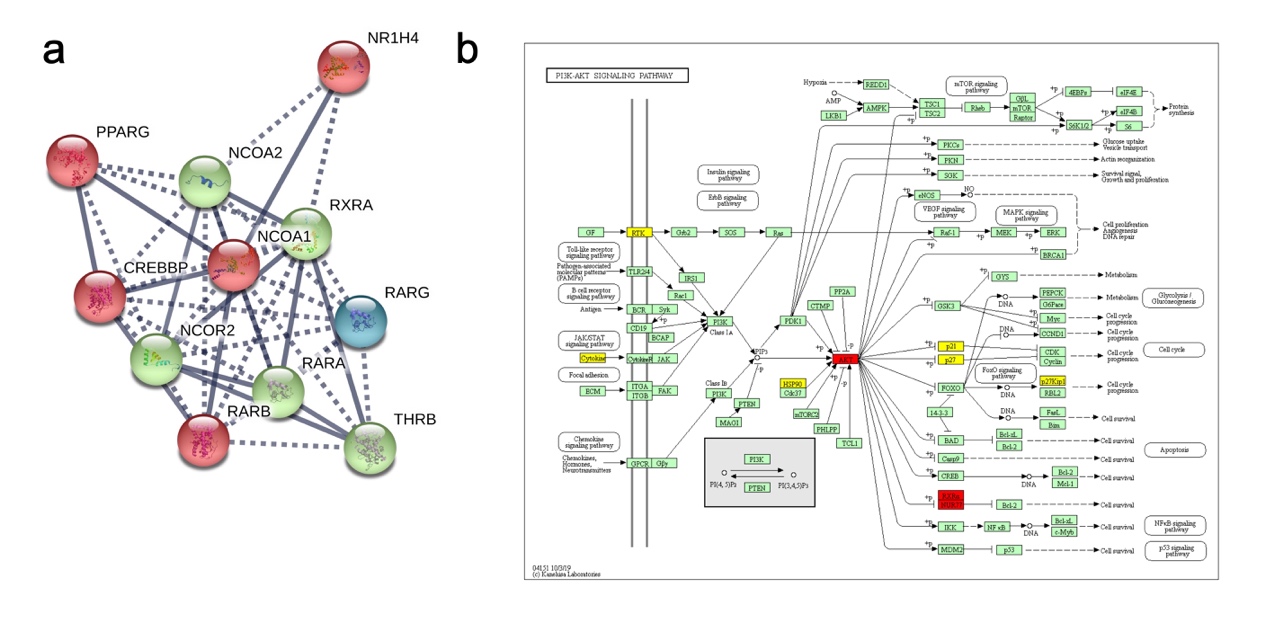
**

**Fig S3. PPI network analysis centered on RXRα.** a: Results of the PPI network analysis centered by RXRα. The minimum required interaction score was 0.9, and the maximum number of interactors to show was 10. The line thickness indicates the strength of the data support, and the results inside the circle represent the protein structure. The network is clustered into 3 clusters and the color represents different cluster. b: Results of the KEGG pathway enrichment analysis of proteins of interest. Red represents the most interesting proteins and yellow represents other CD related proteins.

**Fig S4**


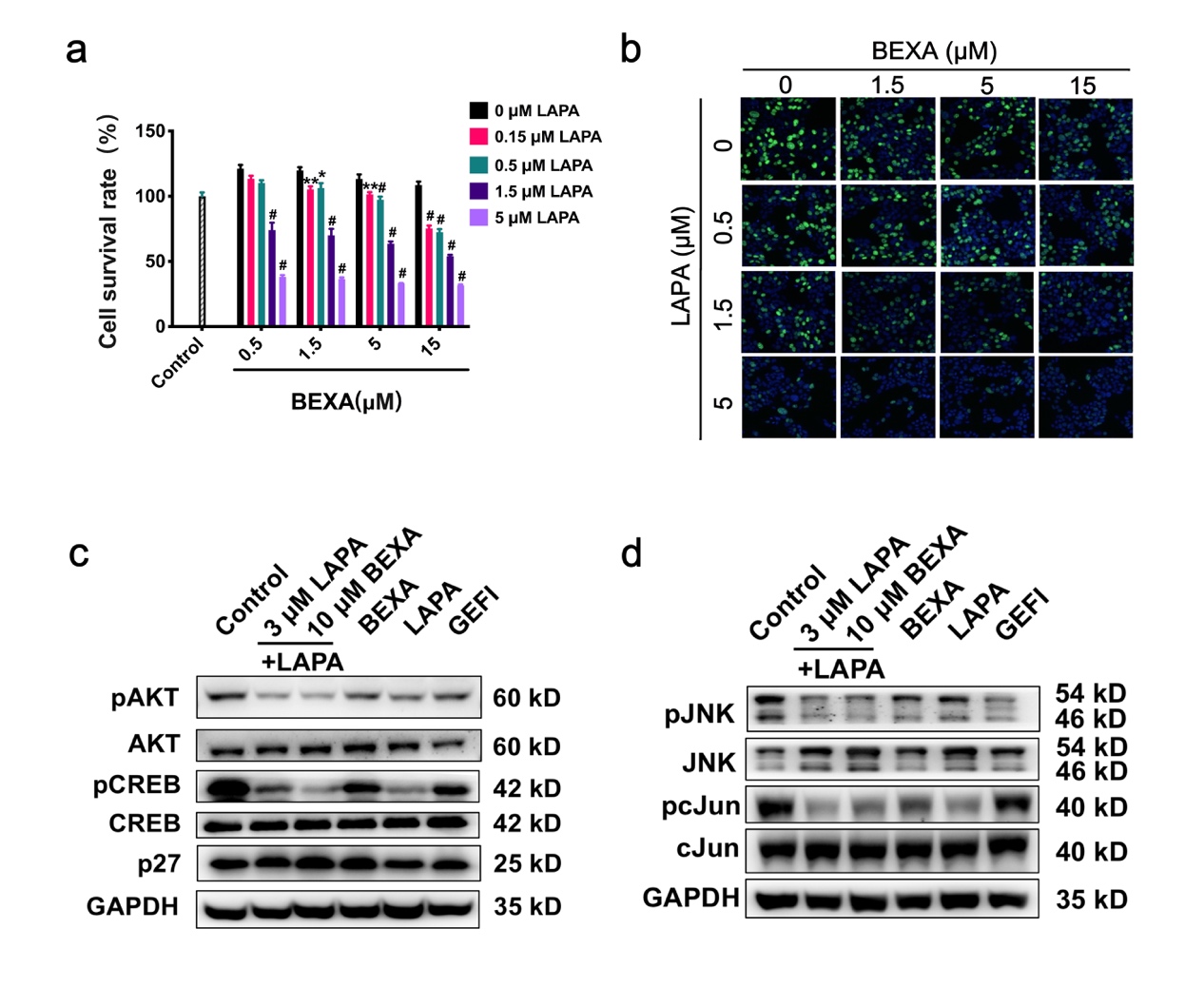


**Fig S4. BEXA and LAPA synergistically inhibit AtT20 cell proliferation via the inactivation of the PI3K-AKT signaling pathway.** **a** AtT-20 cells were seeded onto 96-well plates at a concentration of 2000 cells per well and incubated with BEXA, LAPA or the combination of BEXA/LAPA for next 72 h. Cell survival rate was assessed using the CCK-8 assay according to the ratio of the OD value in cells treated with the indicated drugs to the control cells in three independent experiments with three replicates each. Cells treated with the combined BEXA/LAPA indicated the concentration compared with those treated with BEXA alone, *p<0.05, **p<0.01 and #p<0.001. **b** Quiescent cells were incubated with the indicated drugs for 8 h at the indicating concentrations and then immediately fixed and permeabilized. An EdU incorporation assay was used to detect the DNA incorporation rate. Green fluorescence indicates the positive cells (DNA incorporation) , and DAPI was employed to detect the nuclei. c, d. Representative Western blot images of the expression and phosphorylation of JNK, cJun, Akt, CREB and p27 in AtT-20 cells treated with different drugs for 48 hours. n=3.

**Figure S5**


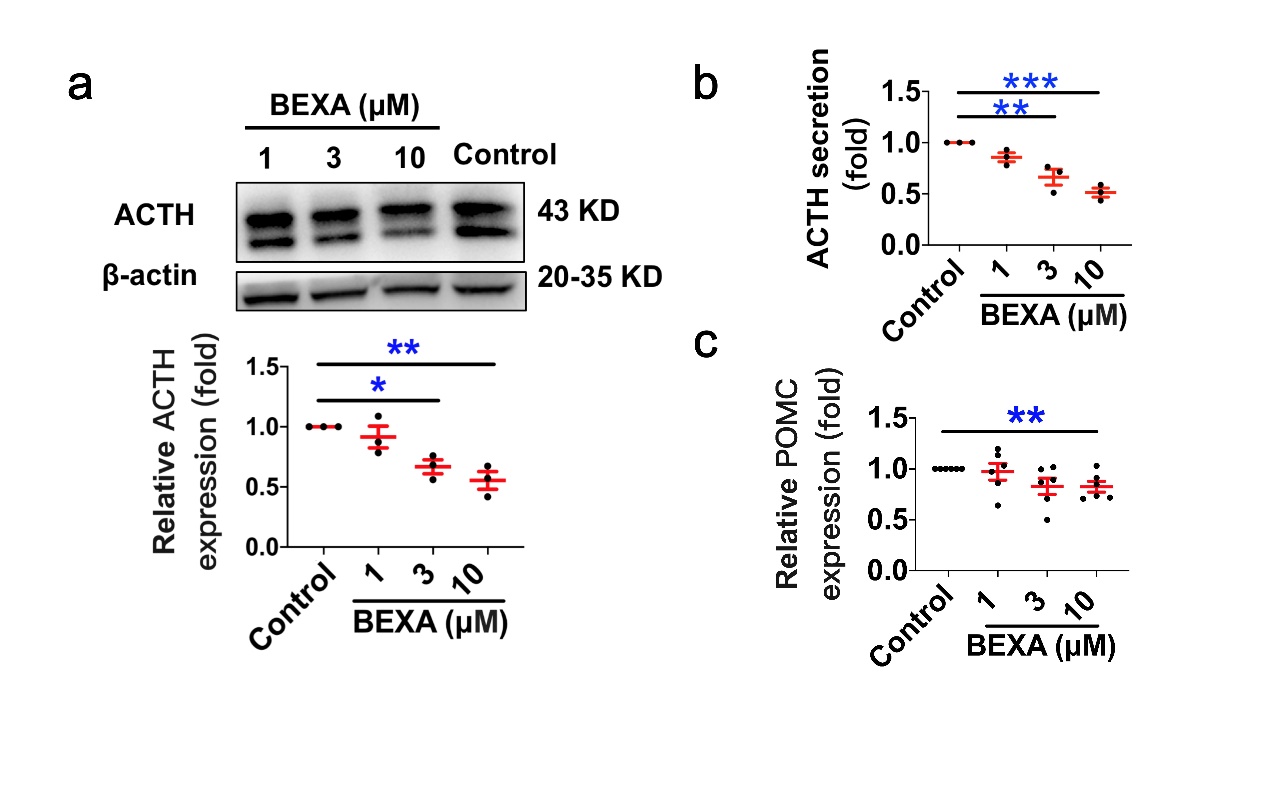


**Fig S5. The ACTH expression and secretion levels and the POMC expression levels in AtT20 cells in response to BEXA in a concentration dependent manner.** a: Cells were seeded in the 100 mm dishes at a concentration of 5×10^5^ cells/dish. After adhering to dish surface (24 h later), AtT20 cells were treated with the indicated concentrations of BEXA, and 48 h later, the expression levels of ACTH in AtT-20 cells and in the supernatant of AtT-20 cells were detected using Western blot (a) and ELISA kit (b) according to manual guide. Additionally, the POMC expression levels were detected using real-time PCR (c). *p<0.05, **p<0.01, ***p<0.001 vs control cells; The experiment was repeated three to four times. Every time, 3 dishes were used in each group. n=3-4.

**Fig S6**

**
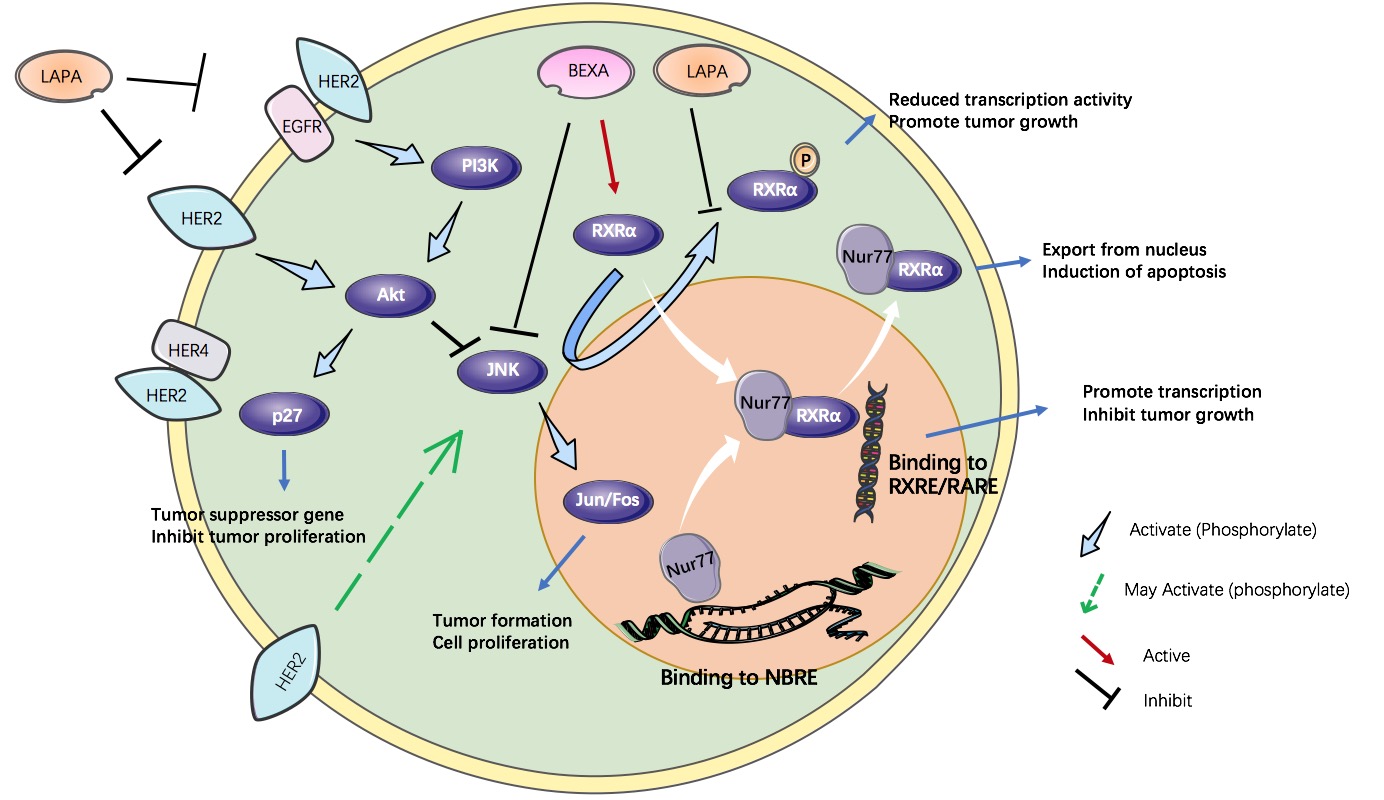
**

**Fig S6.** Mechanism of the synergistic inhibitory effects of LAPA and BEXA on ACTH production and on pituitary adenoma cell proliferation.

**Supplement Tables:**

**Table S1. Top 10 scored hub proteins in Nur77 (NR4A1) PPI network.**

|  | | | | |
| --- | --- | --- | --- | --- |
| Node1 | Node2 | Experimentally Determined Interaction | Database Annotated | Combined Score |
| NR4A1 | RXRA | 0.47 | 0.9 | 0.944 |
| NR4A1 | RXRG | 0.42 | 0.9 | 0.939 |
| NR4A1 | AKT1 | 0.4 | 0.9 | 0.937 |
| NR4A1 | BCL2 | 0.379 | 0.9 | 0.935 |
| NR4A1 | EP300 | 0.379 | 0.9 | 0.935 |
| NR4A1 | MED1 | 0.32 | 0.9 | 0.929 |
| NR4A1 | RXRB | 0.183 | 0.9 | 0.914 |
| NR4A1 | AKT2 | 0.074 | 0.9 | 0.903 |
| NR4A1 | AKT3 | 0.074 | 0.9 | 0.903 |
| NR4A1 | MEF2D | 0 | 0.9 | 0.9 |

PPI: protein-protein interaction.

**Table S2. Approved drugs targeted RXRα.**

| Drugbank ID | Name | Actions | Description | Research Phase for CD Treatment |
| --- | --- | --- | --- | --- |
| DB00459 | Acitretin | Agonist | For the treatment of severe psoriasis in adults. | NA |
| DB00523 | Alitretinoin | Agonist | For topical treatment of cutaneous lesions in patients with AIDS-related Kaposi's sarcoma. | NA |
| DB00307 | Bexarotene | Agonist | Used orally for the treatment of cutaneous T-cell lymphoma (CTCL) | Clinical trial phase 2 |
| DB03756 | Doconexent | Activator | Used as a high-docosahexaenoic acid (DHA) oral supplement | NA |

NA: Not available.

**Table S3. The -docking energy between BEXA and RAR family proteins**

| Gene | RMSD | -Docking energy _original_ | -Docking energy _BEXA_ |
| --- | --- | --- | --- |
| RARα | 0.41 | 61.05 | 8.00 |
| RARβ | 0.31 | 46.72 | 31.71 |
| RARγ | 0.23 | 27.16 | 27.19 |

RMSD: The root-mean-square deviation between the molecular conformations of the docked Ligand1 or Ligand2 and the initial conformations in the crystal structures of RAR α，RAR β or RAR γ.

-Docking energy _original_: The negative docking energy between original ligand and RAR α，RAR β or RAR γ.

-Docking energy _BEXA_: The negative docking energy between BEXA and RAR α，RAR β or RAR γ.

**Table S4. Top 22 scored proteins predicted to interact with BEXA in CD protein network.**

| Gene symbol | Name | Classification | | Effect Model Accuracy | Score |
| --- | --- | --- | --- | --- | --- |
| BRD2 | Bromodomain-containing protein 2 | Transcription/Transcription inhibitor | | 0.889908257 | 99.79366 |
| PPARG | Peroxisome proliferator-activated receptor gamma | Transcription | | 0.787721893 | 99.67229 |
| NR2C2 | Nuclear receptor subfamily 2 group C member 2 | Glucocorticoid receptor | | NA | 99.59137 |
| NR3C1 | Nuclear receptor subfamily 3 group C member 1 | Glucocorticoid receptor | | 0.512237762 | 99.31626 |
| CRH | Corticotropin releasing hormone | Hormone/Hormone receptor | | NA | 97.32573 |
| DRD2 | D (2) dopamine receptor | | Signaling protein/hormone | 0.727065267 | 96.59344 |
| HDAC2 | Histone deacetylase 2 | Hydrolase | | 0.997122302 | 96.24954 |
| MEN1 | Menin | Transcription/Transcription inhibitor | | 0.879310345 | 96.20909 |
| HSP90AA1 | Heat shock protein HSP 90-alpha | | Heat shock proteins | 0.796116505 | 95.48489 |
| PRL | Prolactin | Hormone/Hormone receptor | | NA | 92.26039 |
| AVPR1B | Vasopressin V1b receptor | Signaling protein/hormone | | 0.920634921 | 90.93741 |
|  | Gastric inhibitory polypeptide | Signaling protein/hormone | | 1 | 90.56924 |
| EGFR | Epidermal growth factor receptor | Transferase/Transferase inhibitor | | 0.935976789 | 89.94215 |
| FGFR4 | Fibroblast growth factor receptor 4 | Transferase/transferase inhibitor | | 0.956521739 | 88.14176 |
| EGF | Pro-epidermal growth factor | | Signaling protein/hormone | NA | 87.09795 |
| SMARCA4 | Transcription activator BRG1 | | Transcription/Transcription activator | NA | 86.36971 |
| SSTR2 | Somatostatin receptor type 2 | | Signaling protein/hormone | 0.795640327 | 86.25642 |
| GHSR | Growth hormone secretagogue receptor type 1 | Hormone/Hormone receptor | | 0.569461827 | 85.46345 |
| PRKCD | Protein kinase C delta type | | Signaling protein/hormone | 0.858247423 | 84.99413 |
| SSTR5 | Somatostatin receptor type 5 | | Signaling protein/hormone | 0.87345679 | 84.6907 |
| CRHR1 | Corticotropin-releasing factor receptor 1 | Hormone/Hormone receptor | | 0.744966443 | 84.29421 |
| KLK3 | Prostate-specific antigen | Immune system | | 0.818181818 | 81.67658 |

Note: CD, Cushing’s disease; NA, Not available.

**Table S5. KEGG enrichment analysis of interested genes.**

| Term | Count in gene set | PValue | FDR |
| --- | --- | --- | --- |
| hsa04919: Thyroid hormone signaling pathway | 13 | 9.82E-14 | 1.10E-10 |
| hsa05200: Pathways in cancer | 16 | 1.26E-10 | 1.41E-07 |
| hsa05223: Non-small cell lung cancer | 9 | 2.08E-10 | 2.32E-07 |
| hsa05215: Prostate cancer | 9 | 7.67E-09 | 8.58E-06 |
| hsa05222: Small cell lung cancer | 8 | 1.37E-07 | 1.53E-04 |
| hsa04066: HIF-1 signaling pathway | 8 | 4.02E-07 | 4.50E-04 |
| hsa04024: cAMP signaling pathway | 9 | 3.89E-06 | 4.35E-03 |
| hsa04151: PI3K-Akt signaling pathway | 11 | 4.36E-06 | 4.87E-03 |
| hsa05216: Thyroid cancer | 5 | 1.35E-05 | 1.51E-02 |
| hsa04920: Adipocytokine signaling pathway | 6 | 2.83E-05 | 3.16E-02 |

KEGG: Kyoto Encyclopedia of Genes and Genomes; FDR, false discovery rate.

**Table S6. Drugs targeted EGFR.**

| Drugbank ID | Name | Actions | Description | Research Phase for CD Treatment | IC_50_±SEM |
| --- | --- | --- | --- | --- | --- |
| DB08916 | Afatinib | Inhibitor | For the treatment of metastatic non-small cell lung cancer (NSCLC). | NA | 4.35±0.07 |
| DB12267 | Brigatinib | Inhibitor | For the treatment of patients with ALK+ NSCLC with intolerance to Crizotinib. | NA | 4.95±0.37 |
| DB11963 | Dacomitinib | Inhibitor | Indicated as the first-line treatment of patients with metastatic NSCLC with EGFR exon 19 deletion or exon 21 L858R substitution mutations. | NA | 2.96±0.38 |
| DB00530 | Erlotinib | Antagonist | For the treatment of metastatic NSCLC and pancreatic cancer. | NA | 18.98±3.26 |
| DB00317 | Gefitinib | Antagonist | For the continued treatment of patients with metastatic NSCLC after failure. | Clinical trial phase 2 | 17.63±0.82 |
| DB01259 | Lapatinib | Antagonist | Combine with capecitabine for the treatment of patients with advanced or metastatic breast cancer. | Preclinical trials | 2.67±0.17 |
| DB11828 | Neratinib | Inhibitor | For use as an extended adjuvant treatment in adult patients with breast cancer. | NA | 4.44±0.32 |
| DB09330 | Osimertinib | Inhibitor | For the treatment of patients with metastatic EGFR T790M mutation-positive NSCLC. | NA | 4.82±0.25 |

CD: Cushing’s disease; NA:not available; IC_50_:50% inhibitory concentration; SEM: standard error of mean.

**Table S7. IC50 of bexarotene and lapatinib on Cell survival**

|  | IC_50_ | SEM | N |
| --- | --- | --- | --- |
| Lapatinib | 7.38 | 0.17 | 9 |
| Bexarotene | 96.13 | 1.87 | 9 |

IC_50_: 50% inhibitory concentration; SEM:standard error of mean.

**Table S8. Combination Index of bexarotene and lapatinib.**

| Bexarotene [μM] | CI | | | |
| --- | --- | --- | --- | --- |
|  | + 5μM LAPA | + 1.5μM LAPA | + 0.5μM LAPA | + 0.15μM LAPA |
| 15 | 0.39±0.01 | 0.41±0.01 | 0.46±0.05 | 0.33±0.03 |
| 5 | 0.34±0.00 | 0.47±0.03 | 1.07±0.15 | 2.31±1.30 |
| 1.5 | 0.37±0.02 | 0.89±0.25 | 0.84±0.19 | 1.26±0.76 |
| 0.5 | 0.39±0.02 | 2.25±1.4 | - | - |

CI > 1 is considered to exert antagonistic effect, CI =1 additive effect, CI <1 synergistic effect, and when the CI value is <0.7, it is referred to a significant synergistic effect. n<9 indicates partial data cannot be calculated, “-” indicates all groups of data cannot be calculated.
